# Towards an organisation-wide process-oriented organisation of care: A literature review

**DOI:** 10.1186/1748-5908-6-8

**Published:** 2011-01-19

**Authors:** Leti Vos, Sarah E Chalmers, Michel LA Dückers, Peter P Groenewegen, Cordula Wagner, Godefridus G van Merode

**Affiliations:** 1 NIVEL, Netherlands Institute for Health Services Research, P.O. Box 1568, 3500 BN Utrecht, the Netherlands; 2 Department of Medical Decision Making, Leiden University Medical Center, P.O. Box 9600, 2300 RC Leiden, the Netherlands; 3 NHS Swindon, Thamesdown Drive, Swindon, SN25 2AN, UK; 4 Impact, Dutch knowledge and advice center for post-disaster psychosocial care, P.O. Box 78, 1110 AB Diemen, the Netherlands; 5 Department of Sociology, Department of Human Geography, Utrecht University, P.O. Box 80140, 3508 TC Utrecht, the Netherlands; 6 The EMGO Institute for Health and Care Research (EMGO+), VU University Medical Centre Amsterdam, P.O. Box 7057, 1007 MB Amsterdam, the Netherlands; 7 Care and Public Health Research Institute (CAPHRI), Maastricht University Medical Centre+, P.O. Box 5800, 6202 AZ Maastricht, the Netherlands

## Abstract

**Background:**

Many hospitals have taken actions to make care delivery for specific patient groups more process-oriented, but struggle with the question how to deal with process orientation at hospital level. The aim of this study is to report and discuss the experiences of hospitals with implementing process-oriented organisation designs in order to derive lessons for future transitions and research.

**Methods:**

A literature review of English language articles on organisation-wide process-oriented redesigns, published between January 1998 and May 2009, was performed.

**Results:**

Of 329 abstracts identified, 10 articles were included in the study. These articles described process-oriented redesigns of five hospitals. Four hospitals tried to become process-oriented by the implementation of coordination measures, and one by organisational restructuring. The adoption of the coordination mechanism approach was particularly constrained by the functional structure of hospitals. Other factors that hampered the redesigns in general were the limited applicability of and unfamiliarity with process improvement techniques.

**Conclusions:**

Due to the limitations of the evidence, it is not known which approach, implementation of coordination measures or organisational restructuring (with additional coordination measures), produces the best results in which situation. Therefore, more research is needed. For this research, the use of qualitative methods in addition to quantitative measures is recommended to contribute to a better understanding of preconditions and contingencies for an effective application of approaches to become process-oriented. Hospitals are advised to take the factors for failure described into account and to take suitable actions to counteract these obstacles on their way to become process-oriented organisations.

## Background

During the last decade, hospitals have tried to move from functional towards process-oriented organisational forms. In a process-oriented hospital, the focus is on the process of care instead of on functional departments such as radiology and internal medicine. The central idea of process-oriented organisation design is that organising a hospital around care processes leads to more patient-centred care, cost reductions, and quality improvements [1]. The breakthrough of the process-orientation concept took place at the beginning of the 1990s under the name 'business process reengineering' [1]. Since then, many hospitals have undertaken actions to make care delivery more process-oriented, for example by the implementation of care programmes, clinical pathways, or care pathways for specific patient groups. However, many hospitals struggle with the question of how to deal with process orientation at the hospital level. The realisation of process orientation within the entire hospital organisation demands more of an organisation than performing single projects. Hospitals need to balance the optimisation of care processes with efficiency in use of resources in the functional departments, for example, the use of scarce resources by several patient groups [2].

### Theory

#### Functional organisation design

Traditionally, hospitals have a functional organisation structure. Within this organisational design individuals with a similar area of expertise are grouped into independently controlled departments [1,3-6]. This type of organisation seemed the most appropriate to support and foster the knowledge development required by medical sciences [5]. Departments within a functional organisation design often try to optimise their functioning according to the principles of scientific management. The central thought of scientific management is that efficiency can be improved by the division of labour in such a way that each individual is assigned to a specialised and repetitive activity [7]. However, this task specialisation does not favour the organisation of patients' care trajectories: due to the task specialisation, individual clinicians do not have the capabilities to control the workflow across department boundaries and thus the coordination of the care activities within a patients' care trajectory. The nature of planning in a functional organisation has thus many similarities with that of job-shops that are capacity driven [6,8]. As a result, a complex set of patient flows emerges where the care of the patient, their records, and the resources necessary for care have to be transferred between specialised clinicians and across department boundaries [9]. Bottlenecks occur where one department pushes patients into another department that is not ready to take care of them. Due to this lack of coordination between departments, a functional organisation usually struggles with adaptation and efficiency problems in care processes [9], which in turn affect the quality of care delivery in terms of delivery reliability (*e.g.*, waiting times) [10].

#### Process-oriented organisation design

To improve efficiency and quality of care delivery, it is necessary to overcome the traditional functional organisation structure and reduce the complexity of patients' care processes with its many coordination and transfer points [9]. This can be done by the implementation of a process-oriented organisation design. The central idea of process-oriented organisation can be described as 'structure follows process'; the organisation design is then dominated by cross-functional processes [1,11]. A cross-functional process can be defined as a structured, measured set of activities designed to produce a specific output for a particular customer. It implies a strong emphasis on how work is done within an organisation, in contrast to a focus on what (as in functional organisations) [12].

An important aspect of a process-oriented organisation design is thus that it focuses on the optimal organisation of the process of care instead of functional departments. This means that all different disciplines involved in the care delivery of a patient have to work together as a group and strive to achieve common goals. Ideally, the physical layout is also adapted to the care processes [9,13]. Furthermore, a process-oriented organisation design is characterised by: a less hierarchical organisation, in which people have more responsibility, increased decision making capabilities, and act more autonomously and flexible [14]; less fragmentation of responsibilities by appointing process owners [4,15]; protocols, that ensure smooth coordination, continuity, and less variation between care processes per patient [16,17]; a process-oriented view held by all employees [15]; and performance-based or process-based payments [1,18].

However, there is no such a thing as 'the process-oriented organisation structure.' Process-oriented organisations can have several organisation structures, like a product-line organisation structure [19,20] and a process-based organisation structure [21]. Table 1 outlines the distinctions between functional and process-oriented organisational design.

**Table 1:** Characteristics of functional organisation and process-oriented organisations

	Functional organisation	Process-oriented organisation
Organisation design	Similar capacities are grouped in a department (according to their specialisation, education and training) [1,3], product layout [53]	(a) Similar capacities are grouped in a department (according to their specialisation, education and training) [1,3], product layout [53] with additional coordinating structures (e.g., care programmes) [4]
		- or -
		(b) Multidisciplinary organisational departments which are organised around and based on care processes [1,21], process layout [9,13,53], layout follows process [21]
Organisational Orientation	Vertical orientation [15], objectives for an organisational department can only be linked indirectly to value for the patient [21]	Patient-oriented [21]; horizontal orientation that cuts across the organisational departments [4,21], activities can directly be linked to value for the patients [13,15]
Management focus	Managing departments (pieces of the process) [15], optimising department performance (capacity use) [9]	Managing processes (holistic view) [11,15], optimising patient flow
Decision making	Centralised [11]	Devolved to multidisciplinary teams [21]
Responsibility for care processes	No one is in charge of the processes, because work is organised around tasks [21]	Process owners have the full responsibility for the effective and efficient running of a care process [21]
Coordination between departments	*Ad hoc*, frequent handovers of patients between departments which remain largely uncoordinated [15,54]	(a) Systematic coordination of handovers and co working as rule [54] through additional structural coordination dimensions at the top of the functional structure [21]
		-or-
		(b) Departments have relatively few interdependencies because everyone relevant to the process belongs to the same department, coordination across departments is kept at a minimum [1,21]
Patient flow	Unstructured, unforeseeable and ill-defined [9,15], and therefore a lot of variation in care activities for the same patient groups	Defined [15] and therefore predictable [9], except for clinical exceptions to standardised care processes
Inefficiency costs in care processes	Lots of waste and transfer points resulting in inefficiency costs in the care processes [9]	Lower inefficiency costs in care processes then in functional organisation, because waste and transfer points are reduced [9]

#### Implementation of hospital-wide process orientation

Vera *et al. *[1] and Gemmel *et al. *[1,4] described two main approaches to redesign functional organisation designs to more process-oriented organisation designs -- by implementing coordination mechanisms (*i.e.*, a product line organisation structure or matrix structure) and by organisational restructuring (*i.e.*, a process-based organisation structure).

In the coordination mechanism approach the functional organisation is not changed, but coordinating structures, like care programmes or clinical pathways, are put on top of the existing organisation structure for the realisation of a smooth patient flow across boundaries of hospital departments [4]. These coordinating structures, in the form of lateral connections, are used to bridge barriers erected by an organisation's structure. They establish the sequence of care activities (diagnostics, consultations, treatment) and the responsibilities of professionals involved in the diagnosis and treatment of logistically homogeneous patient groups, *i.e.*, patient groups that need the same type of care activities in the same sequence ('product lines'). As a consequence, everybody involved in the care process should know what to expect in the next, and previous, steps. In the operations management literature methods can be found to assist the establishment of coordination measures aiming to optimise these care processes, such as reengineering [12], lean thinking [13,22] and Six Sigma [23]. These methods describe which steps you should take to set up coordination measures and give ideas for the optimisation of care processes.

In the organisational restructuring approach, the functional organisation is restructured into an organisation with multidisciplinary departments that are based on the needs of a patient ('a process-based organisation structure'). These departments are then composed in such a way that they can handle a care process as comprehensively as possible and have relatively few interdependencies with other departments [1,11,21]. Within the multidisciplinary departments, the tasks are performed autonomously and integratively by cross-functional teams [24]. As a result of this organisational structure, coordination of care processes is facilitated by the elimination of departmental borders, which in turn makes more precise planning possible [25]. However, to reach optimal quality and efficiency, the organisation restructuring is frequently accompanied by the development and implementation of coordination mechanisms.

Several aspects need to be taken into account in the process of organisational restructuring. First, it must be noted that the introduction of multidisciplinary departments must be coherent with a hospital's production structure. It is important to have a minimal critical mass; the multidisciplinary departments need thus to be consistent with the hospital production mix and patients' clinical needs [26]. Second, it is critical to manage and overcome cultural barriers between several medical disciplines. Each medical discipline has its own values, problem-solving approaches, and language (jargon) due to educational experiences and the socialisation process that occur during training of medical professionals [27]. As a consequence, each medical professional primarily identifies with his own professional group, is committed to developing power and prestige of the profession, and looks for professional colleagues for support and censure [1]. These profession-oriented cultures often cause conflict in multidisciplinary teams of process-oriented organisations. Members of multidisciplinary teams frequently experience, for example, role boundary conflicts when team members overstep boundaries of another individual's professional territory [28].

The adoption of either of these approaches does not automatically imply an increase in process orientation [4]. To actually achieve positive effects on efficiency and quality of care, a change of work processes is needed as well. Clinicians, for example, have grown accustomed to working according to particular procedures during years of training and education [29]. These routines are repetitive, recognisable patterns of actions. Routines are confirmed and bound by formal, informal, written or unwritten rules [30,31] like organisational procedures, protocols, and guidelines for care delivery, contracts, agreements, and job descriptions [29]. Adoption of an approach to move towards a process-oriented organisation is a collection of rules as well, which, like other rules, are intended to structure, guide, constitute, allow, oblige, or prohibit particular actions and interactions. However, these new rules are not always followed [31] and its unknown which (combination of) rules are effective.

### Study aim

In an effort to extend the knowledge about transitions towards process orientation at the hospital level, we performed a literature review. The aim of the literature review is to report and discuss approaches that hospitals adopt for the development towards process-oriented organisations and the accompanying factors for success and failure in order to derive lessons for future transitions and research. The scope of this literature review is limited to the process-oriented organisation of clinical processes. Hence, the organisation of management (*e.g.*, organising payments of staff, purchasing goods from suppliers) and ancillary processes (*e.g.*, organising services for cleaning hospital wards and departments) are not taken into account.

## Methods

### Search strategy

We searched the Pubmed, Embase, and Business Source Premier (BSP) databases for relevant English language articles with an abstract from January 1998 through May 2009. This date restriction is based on the fact that hospitals only adopted major redesign plans to become process-oriented organisations since the second half of the 1990s, and results of those plans would reasonably not be available before 1998.

The first step in our literature search was to find useful keywords (MeSH headings) in the Medical Subject Headings database. As a result, we selected six potentially relevant terms: Efficiency, Organisational; Patient-Centered Care; Process Assessment (health care); Organisational Innovation; Product Line Management; Hospital Restructuring. Next, we performed a Major Topic search in Pubmed using these MeSH terms in combination with the MeSH headings Hospitals and Hospital Administration. These two terms were added to the search command because every study had to involve a hospital redesign regarding the management of the internal organisation of the hospital. In Embase, we used the selected MeSH subheadings as keywords in our search. For the search in BSP, the list with all available standard keywords (subjects) in the database was scanned to find useful subjects. We selected 15 potentially relevant terms ('advanced planning & optimisation,' 'advanced planning & scheduling,' 'business logistics,' 'business logistics management,' 'corporate reorganisations,' 'health care reform,' 'organisational change,' 'organisational structure,' 'process optimisation,' 'product lines,' 'product orientation,' 'production engineering,' 'reengineering (Management),' 'work design,' 'workflow'). We searched the BSP database with these keywords in combination with the term 'hospital.'

### Study selection and data extraction

After performing our search with the selected MeSH headings, articles were reviewed on the basis of the title and abstract. The studies had to assess hospital redesign that aimed to improve the control of at least two interfering care processes in terms of process-related topics. The studied redesigns should not (mainly) be aimed at changing the specifics of clinical practice, but should concern improvements in the flow of patients through the hospital. Inclusion and exclusion criteria are summarised in Table 2. We decided not to specify inclusion criteria on outcome measures too strictly beforehand. Process orientation is a broad concept, covering a variety of structure, process, and outcome parameters. Furthermore, we did not set criteria for study designs used for the evaluation of the redesigns towards process-oriented organisations. In order to understand and evaluate this kind of intervention, research methods need to shed light on the interaction between the intervention and its context [32]. Therefore, studies using observational research methods are also included in this study next to quantitative methods.

**Table 2:** Inclusion and exclusion criteria literature review

Inclusion criteria	Exclusion criteria
Article should:	Article focuses on:
- Contain an abstract;	- Staff satisfaction and/or change only concerns job redesign or responsibility changes;
- Be written in English;	- Changing the organisational structure or redesigning at organisational level without aiming improvement of patient flow;
- Focus on hospital organisations;	- Changing the health structures at national levels;
- Address a restructure or redesign of patient flow at organisational level, or at least for two interfering care processes;	- Changing hospital ownership or affiliation;
- Contain a description of the transformation process/actual intervention;	- Projects with main purpose of financial improvement, except where this is used to form basis of organisational change or incentives;
- Be a study and not an editorial, letter to the editor, or opinion piece;	- Changing the organisation of a single functional unit or a single care pathway;
- Have been published after 1 January 1998 and before 1 May 2009.	- Change in software and/or hardware and IT with no intended effect on patients flows;
	- Description of methods, model and theories without empirical data;
	- The management of redesign and change projects;
	- Redesign of buildings.

Two reviewers (LV and SC) independently scanned titles and abstracts to select studies for consideration. Initial disagreements on study selection were resolved reaching consensus. Publications were selected for further assessment of the full text if inclusion criteria were met or if it was impossible to determine this based on the abstract. We used a standardised extraction checklist to obtain data on the main characteristics of the redesigns, study design, approaches used, relevant results, and factors for success and failure. Further, we looked in particular whether hospitals undertook specific measures to promote the adoption of new rules of the process-oriented organisation design within working procedures.

Additionally, we performed an extra search on the internet using Google^® ^to find additional information about the redesigns that were described in the included articles of our search. For this search we used the name of the hospital and the keywords 'redesign' and 'reengineering.'

## Results

**Figure 1:**
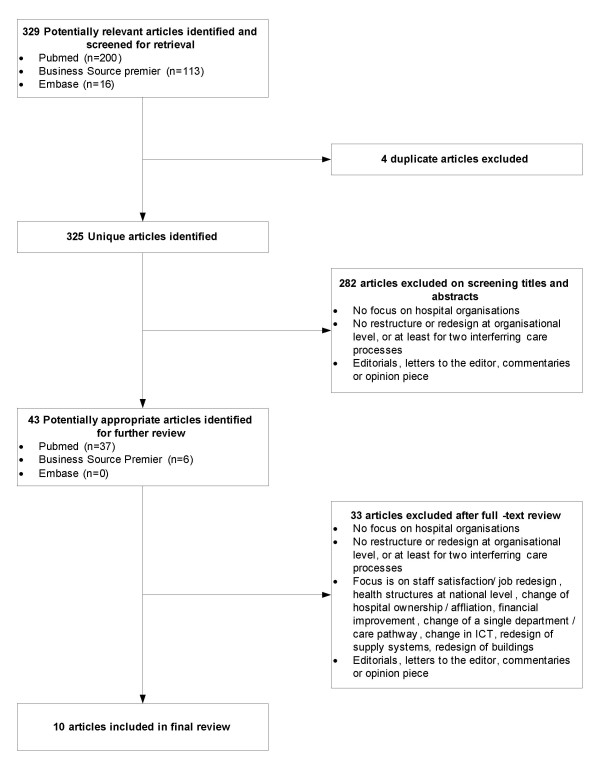
**Selection process for studies included in analysis**.

Figure 1 shows the flow of papers through the review. Overall, 325 abstracts of articles published between January 1998 and May 2009 were identified. During abstract screening, 282 articles were excluded because they did not meet the inclusion criteria. A total of 43 articles was selected for detailed review, 33 additional articles were excluded subsequently for not meeting inclusion and exclusion criteria. Three of the ten remaining articles described different aspects of the redesign of Policlinico A. Gemelli (PG) [33-35], and two other articles described different aspects of the redesign of the Leicester Royal Infirmary (LRI) [36,37]. The remaining four articles described redesigns of Denver Health (DH), Flinders Medical Center (FMC) and University of Wisconsin Hospitals and Clinics (UWHC). As a result, a total of five redesigns are described in this review. Our search on the internet using Google^® ^provided extra information about the redesigns of DH [38], FMC [39-41] and LRI [42,43].

The study designs, approaches used, applied supporting measures for the adoption of the approach, reported outcomes, and factors for success and faced challenges of the five included redesigns are summarised in Table 3, 4, and 5 based on the retrieved literature.

**Table 3:** Overview of included redesigns

	Denver Health (DH)	Flinders Medical Center (FMC)
Setting	A 398-bed hospital in Denver, United States	A 500-bed teaching general hospital in Adelaide, Australia
Aim redesign	To improve patient safety and satisfaction, efficiencies and cost reductions, and job satisfaction	To improve patient flow through the emergency department (ED), medical and surgical patients
Study design	Uncontrolled before-after study, including an analysis of positive and negative antecedent conditions	Uncontrolled before-after study
Evaluation period	2003 to 2008	2003 to 2007
Redesigned services	Clinical care and administrative processes	Clinical care (first emergency care, then surgical care, medical care)
Applied approach	Coordination mechanism approach	Coordination mechanism approach
Measures to change working procedures	Not reported	Not reported
Outcomes in general	Reductions in operating room expenses; fewer dropped patient calls; cost savings	Positive results for redesign at the emergency department (less congestion; reduced throughput time); No outcomes reported for the elective surgical care process redesign
Outcomes on indicators		
Finances	No quantitative figures reported	No quantitative figures reported
Operational efficiency	No quantitative figures reported	Length of stay:
		- Time spent at the ED: ↓ (from 5.4 hours to 4.8 hours).
		- Length of stay of emergency admissions: ↓ by one day.
		Throughput time:
		- The number of patients leaving the ED without waiting to be treated: ↓ (approximately from 4% to less than 2%)
		Patient volume:
		- Patients seen at the ED: ↑ (from 140 to a range of 180 to 210 patients per day [managed within the same physical space and with similar staff-patient ratios]).
		- Emergency admissions: ↑ (from 1,200 to over 1,600 a month).
Patient Satisfaction	No quantitative figures reported	No quantitative figures reported
Patient Safety	No quantitative figures reported	Adverse events:
		- Number and types of serious adverse advents throughout the hospital a year: ↓ (from 91 to 19)
Factors for success	The change strategy was built on ideas that were developed and tested in preceding projects; Leader of transformation was a clinician, who drew on her professional status and familiarity with clinical practice; Political and financial support of the city; Training of nurses, clinicians and middle managers in Lean improvement techniques; Previous (positive) experience with change management	Leadership by senior executives; Clinical leadership; Team-based problem solving; A focus on patient journey; Access to data; Ambitious targets; External facilitators to break down the 'silo' mentality and facilitating multidisciplinary teamwork; Organisational readiness; Selection of projects - start the redesign process with a problem that obviously needs to be fixed; Strong performance management; A process for maintaining improvement; Communicating the methodology and results in many different ways, i.e., Lean thinking days
Challenges	To manage system-wide changes while horizontal communication across occupations, departments and sites is impeded; To promote the use of industrial techniques to clinicians while they lack experience working with these problem solving and quality improvement techniques; To manage shortcomings in IT infrastructure in providing data for RIEs; To mobilise (financial) resources needed for the redesign while the hospital has safety net obligations (cannot delete services)	To manage the tension between the bottom-up approach of Redesigning Care and the more usual 'command and control' (top-down) process adopted by healthcare managers who, once the problem is identified, see their role as coming up with a solution that front-line staff then have to implement

**Table 4:** Overview of included redesigns, continued

	Leicester Royal Infirmary (LRI)	Policlinico A. Gemelli (PG)
Setting	A > 1,000-bed university hospital in Leicester, United Kingdom	A 1,500-bed teaching hospital in Roma, Italy
Aim redesign	To improve hospital performance in all areas (including hospital costs, patient process times, length of in-hospital stay) dramatically	To introduce a new patient-oriented mentality; to reduce costs
Study design	Uncontrolled before-after study and a process evaluation	Uncontrolled before-after study
Evaluation period	1995 to 1998	1995 to 1998
Redesigned services	All patient services (outpatients' and clinical care)	All patient services (outpatients' and clinical care)
Applied approach	Coordination mechanism approach	Coordination mechanism approach
Measures to change working procedures	Process management	Not reported
Outcomes in general	The impact of redesign on hospital services, costs and organisation was not as dramatic as initially anticipated (initial targets were ambitious); The overall efficiency was not transformed (as assessed through a quantitative evaluation of its performance)	Positive results for the introduction of the DC and reorganisation of surgical wards; Results of the medical wards are positive but have to be further improved to reach goals of the redesign
Outcomes on indicators		
Finances	Output per £ (in comparison with other teaching Trusts), some examples:	No quantitative figures reported
	- Weighted activity per £ of operating costs: ↑ (from £44 million to £55 million cheaper than average).	
	- Weighted activity per staff numbers (staff productivity): ↑ (from 21% to 41% better than average).	
	N.B. At macro level it is not possible to directly attribute the efficiency improvements to re-engineering - a number of other driving forces were also having influence.	
Operational efficiency	LRI used a lot of measures, some examples:	Length of stay:
	- Length of stay: ↓ (from 4.93 to 4.68)	- Preoperative hospital stay of surgical patients: ↓ (from 57 to 4.1 days)
	- Bed throughput: ↑ (from 66 to 78).	- Preoperative hospital stay of medical patients: ↓ (from 10 to 9.6 days).
	- Total admissions per bed (a year): ↑ (89 to 108)	
	- Percentage of bed occupancy: remained stable around 80%	
Patient Satisfaction	Patient satisfaction surveys among 'walking wounded' patients: no change	No quantitative figures reported
Patient Safety	No quantitative figures reported	No quantitative figures reported
Factors for success	Not reported	Not reported
Challenges	To mobilise enough commitment to reengineer while clinical involvement in laboratories was low; To ignore the need for tailoring of interventions to clinical situations; To manage divergent views about nature and purpose of services between reengineers and clinicians; To manage changes that crossed specialty and directorate boundaries; To have the right ambition (results may not be at expense of learning or generate cynicism instead of interest and enthusiasm)	To manage changes that involve more hospital departments. For example, in surgical wards, the activity as a whole is conditioned by the operating rooms, while in medical wards, functioning is very complex and interacts with the entire hospital

**Table 5:** Overview of included redesigns, continued

	University of Wisconsin Hospitals and Clinics (UWHC)
Setting	A 489-bed tertiary care centre in Madison, United States
Aim redesign	To improve efficiency and patient satisfaction, and stabilising institutional financial health while keeping quality high
Study design	Uncontrolled before - after study
Evaluation period	2000 to 2004
Redesigned services	Heart and vascular care, oncology and paediatric care
Strategy type	Organisational restructuring approach
Measures to change working procedures	Incentives for clinical care lines and departments
Outcomes in general	Financial: each clinical care line demonstrated improved percent margin, improved net revenues, and increases in local and regional market share; Operational: operational efficiency, measured by patient volume change, inpatient length of stay data, improved from pre clinical care line metrics; Patient satisfaction: improved patients satisfaction surveys were documented for each clinical care line
Outcomes on indicators	
Financial	Margins (profits [%]): - Heart and vascular care: ↑ (from 4.2 to 10.3) - Oncology: ↑ (from 14 to 15.5) - Pediatric care: ↑ (from -8.2 to -0.8 )
Operational efficiency	Length of stay: - Heart and vascular care: ↓ (from 8.5 to 5.5 days) - Oncology: ↓ (from 6.7 to 6.0 days) - Pediatric care: ↓ (from 5.4 to 4.4 days) Patient volume (Inpatients discharges [ID]/outpatients visits [OV]): - Heart and vascular care: ID ↑ (from 3220 to 3550), OV ↑ (from 31.915 to 36.556) - Oncology: : ID ↑ (from 2738 to 2795), OV ↑ (from 87.858 to 89.507) - Pediatric care: : ID ↑ (from 2632 to 3047), OV ↑ (from 114.369 to 123.997)
Patient Satisfaction	Press Ganey Surveys for overall rating of care received: - Heart and vascular care: ↑ (from 85 to 96) - Oncology: ↑ (from 85 to 94) - Pediatric care: ↑ (from 85 to 91)
Patient Safety	No quantitative figures reported.
Factors for success	Enthusiastic participation of clinicians and their willingness to change practice patterns to achieve care efficiencies; Administrative support which made it possible to reorganise and relocate care units within the hospital to centralise areas of specialty care and to adopt universal nursing practices on units where patients had similar requirements
Challenges	To get agreement for collaboration of staff clinicians and their willingness to change practice patterns

### Main characteristics of redesigns

The articles reported on redesigns in Australia (FMC), Italy (PG), United Kingdom (LRI) and United States (DH, UWHC) [33-37,44-46]. Two of these redesigns aimed to implement process orientation for all patient services, including outpatients' and clinical care (PG, LRI) [33-37]. The other redesigns were limited to clinical care (DH, FMC) [44,45] and three clinical care lines (heart and vascular care, oncology and paediatric care) (UWHC) [46]. All redesigns aimed to improve the patient flow through the hospital. Some redesigns had additional goals: cost reductions/efficiency improvements [33-37,45,46], patient safety [45], patient satisfaction [45,46], and job satisfaction [45].

### Study designs

All redesigns were evaluated in uncontrolled before-after study designs. From the assessment of the PG, DH and FMC redesigns, precise information on study design, data gathering strategies, and outcome measures were lacking. The evaluation of the LRI redesign contained an assessment of changes in quantity and costs of the healthcare delivered using routine hospital and health authority data sources and specific monitoring data of the redesign programme [43]. Besides, a process evaluation that aimed to describe antecedents, context, implementation, and impact of the LRI redesign, and to derive lessons regarding management of change, was performed [43]. For this process evaluation, additional qualitative data were gathered by documentation research, interviews, and notes from informal conversations and observational data from meetings. The evaluation of the UWHC redesign included service-line metrics on financial performance, operational efficiency, and patient satisfaction using hospital data and patient surveys [46].

### Approaches used to move towards a process-oriented organisation

#### Coordination mechanism approach

Four of the five redesigns (DH, FMC, LRI, and PG) followed the coordination mechanism approach for the implementation of process orientation. Three of these redesigns (DH, LRI and PG) identified first common processing steps in medical treatment processes of patients, *e.g.*, triage, diagnosis, and treatment. They subsequently analysed and optimised these processing steps by implementing coordination measures.

DH selected five overarching processing steps, 'access,' 'inpatient flow,' 'outpatient flow,' 'operating room flow,' and 'billing' as targets for the redesign of clinical care and administrative processes [38,45]. For each processing step, a detailed map was created to diagram its current state, ideal state, and likely future state. DH then initiated a series of week-long 'Rapid-Improvement Events (RIEs),' five of which were conducted each month to improve individual processes within each processing step. In these RIEs, processes were mapped and unnecessary activities removed. For example, a RIE for the processing step 'access' was to improve the telephone call abandonment rate. Next to the optimisation of common processing steps, DH focused on development of its infrastructure for information technology and workforce (identifying the 'right people' through personnel selection techniques).

LRI identified four hospital processing steps, 'patient visit,' 'patient test,' 'emergency entry,' and 'hospital stay,' and planned to redesign these processing steps within specially created 'laboratories' [36,37]. Originally, they planned to redesign the 'patient test' and 'patient visit' (diagnostic services and outpatient clinics) first before redesigning 'emergency access' and 'patient stay' (clinical care processes). However, this phased approach was replaced by plans to redesign the processing steps concurrently to reduce chances of creating a partially redesigned organisation and to manage the interaction between hospital processes and challenging existing departmental and functional boundaries. Nevertheless, reengineering became more local than corporate because it was shaped and managed at the level of groupings of functional departments. The 'laboratories' were dismantled and the responsibility and accountability for redesign projects were shifted from reengineers in laboratories to functional departments to better suit the redesign of the processing steps to local interests and agendas.

PG identified five processing steps of the medical treatment process of patients as targets for their redesign: 'emergency care,' 'outpatient care,' 'diagnostic service and laboratories,' 'operating rooms' and 'medical/surgical care' [33,34]. Subsequently, PG identified patient groups that are processed equally within these processing steps, *e.g.*, outpatients or inpatients that are booking an outpatient (follow-up) appointment. Next, they optimised these processing steps, starting at the pre-hospitalisation process and the scheduling for outpatients appointments. The pre-hospitalisation process was, for example, optimised by planning all preoperative care activities (routine tests, initial patient evaluation) on one day.

In contrast to the three redesigns described, FMC did not focus its redesign at the optimisation of individual processing steps of care processes (*e.g.*, scheduling outpatients' appointments), but on the optimisation of the patient flow between and within processing steps of care processes [39,44]. FMC first divided the clinical care processes in emergency, surgical, and medical care. Within these three groups, FMC identified high volume patient flows by searching for patient groups that had a number of processing steps in common ('patient-care families'), for example for 'short emergency care' (likely to be discharged) and for 'long emergency care' (likely to be admitted). Next, they looked at the processing steps of the identified patient-care families to improve the sequencing of the processes involved by eliminating 'waste': steps in a care process that do not add value to a care process (*e.g.*, waiting times, unnecessary movement of personnel and patients). This involved mapping out the daily processes for clinical teams, then obtaining agreement on new sequences. Once an efficient and effective way of undertaking a process had been developed and agreed on, it became standard procedure. This happened for instance for the way medical staff organise their day across the hospital [39,44]. While using this method, FMC worked gradually towards process orientation of their clinical care processes: first, they redesigned all emergency care processes, followed by the surgical and medical care processes.

#### Organisational restructuring approach

UWHC followed the organisational restructuring approach. UWHC gradually worked towards a clinical care line matrix structure, in which disease-and patient-based processes are streamlined in focused clinical units. An internal and external market analysis led to the selection of the first three clinical areas (heart and vascular care, oncology, and paediatric care) for clinical care line development [46]. These three areas had the necessary leadership in place, institutional strength, and there was regional need for these services. The services were centralised to geographical areas of the hospital dedicated to care and management of these patient groups. This included relocation and redesign of hospital units and diagnostic facilities for heart and vascular patients, the oncology clinical care line, and the construction of a free-standing adjacent children's hospital tower [46]. In 2006, UWHC was planning to expand from three to six clinical care lines. The newest additions were transplantation, neuroscience, and orthopaedics.

### Supporting measures to change working procedures

It appeared that two hospitals took supporting measures to promote compliance to the rules of the process-oriented organisation design on the work floor. Within the redesign of LRI, hospital management tried to enforce compliance by changing authority and power structures. LRI introduced process management as an attempt to strengthen managerial accountability and responsibility for patient processes at the level of the functional departments, and to improve managerial communication and decision making across functional departments [36,37]. UWHC developed an incentivisation process that allowed both departments and clinical care lines to have financial rewards for success in order to enforce compliance to the new working methods as well as to sustain the quality of all services that were not yet redesigned [46].

### Reported outcomes of the redesigns

There are large differences between the types of outcomes described. Of four redesigns (FMC, PG, LRI, and UWHC) data from before and after implementing changes to become process-oriented were reported (Tables 3, 4, and 5) [34,44,46]. The reported results of the FMC and PG redesigns were limited to a number of positive outcomes on operational efficiency for specific patient groups or specific departments (*e.g.*, throughput times and length of in-hospital stay) [34,44]. LRI and UWHC reported results on financial outcomes, operational efficiency, and patient satisfaction. LRI's redesign led to improvements on financial indicators and indicators for operational efficiency, but these were not as big as initially anticipated. It appeared that improvements in the individual sectors of the hospital only produced a marginal improvement in the overall efficiency of LRI [36,37]. Furthermore, LRI did not succeed in significantly reconfiguring previous patterns of organisation: clinical directorates and specialties survived as organisational forms [37]. The redesign of UWHC resulted in improved operational efficiency, patient satisfaction, and financial performance [46]. Of the remaining redesign, DH, only qualitative descriptions of the results were reported in the retrieved literature: 'it led to reductions in operating room expenses, fewer dropped patient calls and cost savings' [45].

### Factors for success and challenges faced

In three redesigns (FMC, DH and UWHC), we found factors for success in the retrieved literature, including: senior management support [41]; clinical leadership and involvement [41,45,46]; team-based problem solving [41]; adequate Information and Communication Technology (ICT) support [41,45]; administrative support [46]; ambitious targets [41]; external facilitators [41]; organisational readiness [41]; selection and execution of projects in order of urgency [41]; using a change strategy that already proved to be successful [45]; and good communication and training in the quality improvement techniques [41,45].

In the retrieved literature about all five redesigns challenges to the redesigns were reported (Tables 3, 4, and 5). The main challenges that were reported by the hospitals that followed the organisational restructuring approach were related to the improvement techniques used within the redesigns, the organisational structure, and the nature of care delivery. Three of the four hospitals (FMC, DH, and LRI) mentioned that the technique used for process improvement was sometimes challenging. Two of these hospitals made use of 'lean' as core technique, which originates from industry. The aim of this technique is to optimise care processes or processing steps by the elimination of activities that do not add value to the patients, like waiting times or movements of staff and patients. In DH, the application of 'lean' was sometimes difficult because clinicians lack experience with this kind of improvement technique [45]. In FMC, the 'lean' technique posed a challenge to the middle and senior managers [44]. They had to change roles from the traditional, top down, problem-solving responsibilities towards a more bottom-up approach, in which they first had to understand how the work is done as well, as what the root causes of delays are and other impediments to flow, before they could come up with a solution. In LRI, the redesign was based on business process redesign, which aims at radical improvements. Consistent with the logic that people need to think big and radically to realise big improvements, LRI set ambitious aims for its redesign, but these turned out to be too ambitious, which came at the expense of learning and generated cynicism instead of interest and enthusiasm [42].

Furthermore, two hospitals (LRI and DH) reported that the nature of care delivery prevented them to fully apply the selected approach to come to a process-oriented organisation. DH, did not feel free, like most firms in industry, to delete services and focus on strategically important services [45]. This hampered DH to free financial resources needed for the redesign. In LRI, the nature of care delivery hampered the hospital to adopt the standardised hospital processing steps 'patient visits,' 'patient test,' 'emergency entry,' and 'hospital stay' to every patient group in a rapid and mechanistic fashion. The need to tailor the redesigned processes to different patient groups took time.

In addition, three hospitals (PG, DH, and LRI) reported that the existing departmental and functional boundaries hampered the implementation of the redesign. PG experienced that making changes was much more difficult in departments that interact with the entire hospital -- *e.g.*, the operating room -- than in departments that only depend on input of one department, such as surgical wards [34]. DH perceived a lack of horizontal communication across occupations, departments, and sites [45]. LRI experienced that making changes across the interfaces of existing specialties and clinical directorates was a slow and difficult process. The introduction of process management to improve managerial communication and decision making across specialties and clinical directorates could not significantly change this pattern [37].

In contrast, the hospital that adopted the organisational restructuring approach did not report any of the abovementioned difficulties. Instead, UWHC reported difficulties in clinician collaboration [46].

## Discussion

Worldwide, hospital organisations are changing their functional structure into structures that focus on patient care processes. In this literature review, we assessed five examples of hospitals that pursued a process-oriented organisational form and the accompanying factors affecting their success or failure in the redesign process. The study points out that four out of five hospitals tried to move to a process-oriented organisation of care by the implementation of coordination mechanisms. Only one of them followed the organisational restructuring approach. From the results of these hospitals, it seems that the adoption of either approach can possibly lead to the desired process orientation. The UWHC redesign demonstrated that the adoption of the organisational restructuring approach can be relatively successful: patient satisfaction, financial outcomes, and operational outcomes of the redesigned services were improved. However, the UWHC adopted the organisational restructuring approach for only three, and later on six, strategically important patient groups. This leaves the question whether the organisational restructuring approach would also be potentially successful for strategically less important services or for the organisation of care delivery for patients with needs that do not fit within existing clinical care lines. Vera *et al. *already pointed out that this could be difficult, because political and ethical obligations of hospitals prevent them from withdrawing small volume services to focus only on strategically important ones of sufficient volumes that justify the creation of multidisciplinary departments [1].

Three of the four other hospitals (DH, FMC, and PG) demonstrated that the coordination mechanism approach can lead to positive results, but they did not report on the results very extensively. FMC and PG only reported some general results on process measures, and DH only reported qualitative descriptions. LRI, on the other hand, evaluated its redesign extensively, but the results were disappointing: financial outcomes and practice patterns showed no improvement. Patient satisfaction was not measured. From the reported factors for failure, it appeared that the adoption of the coordination mechanism approach was constrained, particularly by the functional organisation design of hospitals. Improvement of control at process level by coordination measures requires that departments give priority to the service level provided to patients (*e.g.*, short access times, waiting times for diagnostic examinations, and throughput times) instead of to the performance of their department (utilisation of resources) [47]. An attempt of LRI to break the previous pattern of the functional organisation by the implementation of non committal process management did not work [37,42]. This underlines the importance of measures that enforce compliance. Vera *et al. *recommends, for example, the establishment of financial incentives that are based on the performance of care processes [1]. Further, it seems that within this approach an initial focus on logistically homogeneous patient groups, assisted by bottom-up knowledge of healthcare professionals, could help to overcome the functional division of labour. The redesigns of DH, LRI, and PG did not focus on care processes of logistically homogeneous patient groups but on single steps of care processes. This resulted only in improvements to the processes of individual departments instead of changes improving the overall coordination of care activities, delivered by several departments within a patients' care process. Optimisation of these 'isolated' processing steps did not lead to more collaboration between departments and more process orientation within the whole care trajectory of patients. Besides, the optimisation of linkages between the processing steps did not get the attention of the hospital because of the focus on 'isolated' processing steps. However, it appeared from the FMC redesign that an initial focus on logistically homogenous patient groups stimulated encouraged healthcare professionals to work together as a group to optimise linkages between processing steps, and to delete all steps in a care process that did not add value.

Next to these specific points of interest for the different approaches to become process-oriented, we could derive some more general lessons for future redesigns from the results of the literature review. First, tailoring is needed. LRI tried to rollout general redesigns of processing steps to every clinical situation, but this appeared to be impossible due to the multitude of different clinical and disease patterns. Second, clinical engagement and additional administrative support for the use of quality improvement techniques is crucial to the success of the redesign. The evaluations of the DH, FMC, LRI, and UWHC redesigns pointed out that changes to clinical services cannot succeed without the input of clinicians. On the other hand, it appeared in two redesigns (FMC and DH) that clinicians lack experience in applying improvement techniques. Besides, professionals working in hospitals already face huge demands on their time, and, justifiably, may not always be willing to prioritise time consuming service redesign over spending time with patients. Therefore, it is very important to involve clinicians in redesigning services and to also offer them administrative support with the development and the implementation of coordination measures.

Unfortunately, we are not able to judge which of the two approaches delivers the best results in which circumstances. For such an assessment more studies are needed. Such studies have to include information on study design, objectives, approach, patient population, and results.

Limitations of the review should be considered in interpreting the results. Due to the limited number of studies found, the generalisations made are weak. As in any review, we may have missed relevant studies. We believe that given the worldwide amount of activity of hospitals to become process-oriented, a very limited number of studies have been published addressing approaches to move towards a process-oriented organisation design. This is probably due to the nature of the phenomenon studied. Like other types of planned change or innovation, (successful) organisation wide redesign moves sequentially, from awareness of gaps to identification of solutions, implementation of selected solutions, and institutionalisation of solutions [20]. This hampers evaluation and publication of these kinds of interventions. Another explanation of the limited number of studies we found could be the fact that process orientation in hospitals does not succeed and that studies about failures are not published. The cause of failure could be the strong institutionalised functional division of tasks in healthcare systems, which also exist within the education of medical professionals.

To extend theories and knowledge about the best approaches to become process-oriented, and how to overcome barriers to success, it is important that the main focus of future research is on the preconditions or contingencies for an effective application of process-oriented organisation designs in healthcare, and how to ensure appropriate application at a later date. To gain insight into these preconditions and contingencies, we recommend using qualitative research methods (*e.g.*, observation, semi-structured interviews) as well as quantitative methods. Qualitative research methods are able to shed light on the interaction between the characteristics of the redesign and its environmental and organisational context, which cannot be clarified by quantitative research methods [48-51]. This is important because redesigns are complex interventions that are introduced into complex 'social worlds' [50,52].

Further, it is important that future research focuses on the development of a valid measure for the degree of process orientation that allows us to compare results of several redesign initiatives. Vera *et al. *[1] and also Gemmel *et al. *[4] have already made the first steps for the development of such a measure. However, their measures do have their limitations. The items used (*e.g.*, clinical pathways, multidisciplinary teamwork, performance-based payment) to measure process orientation by Vera *et al. *were quite global so that responding to these items for a whole hospital leaves a great deal of room for measurement error [1]. The measure used by Gemmel *et al. *did not include all dimensions of process orientation, but did focus on the process-oriented of medical professionals [4]. However, the degree of process orientation of medical professionals is very important in determining the success of implementation: after all, top management may change structures, including reporting responsibilities of middle and lower management, but this does not automatically lead to more process orientation in work processes, which is needed to effectuate the process orientation at hospital level.

Next, future research needs to develop a set of valid measures for outcomes of redesigns to be able to determine and compare results of redesigns. This set should consist of indicators for financial outcomes, operational efficiency, medical outcomes, patient satisfaction, and patient safety.

## Conclusions

Due to the limitations of the evidence, it is not known which approach, implementation of coordination measures or organisational restructuring (with additional coordination measures), produces the best results in which situation. Therefore, more research is needed. Within this research, the use of qualitative methods in addition to quantitative measures is recommended to be able to understand the interaction between the characteristics of the redesigns and their context. Hospitals are advised to take the factors for failure described into account and to take suitable actions to counteract these obstacles on their way to become process-oriented organisations.

## Competing interests

The authors declare that they have no competing interests.

## Authors' contributions

LV was responsible for designing the study, conducting the literature review, analysing and interpreting the data, and drafting the manuscript. SC assisted LV in designing the study, conducting the literature review and drafting the manuscript. MD assisted in interpreting the results and drafting the manuscript. PG, CW and GM participated in the design of the study, assisted in interpreting the results, the critical revision of the manuscript and its supervision. All authors have read and approved the final manuscript.
